# Turkish adaptation study of the Metacognition Assessment Scale-Abbreviated in psychotic disorders

**DOI:** 10.3389/fpsyg.2026.1884522

**Published:** 2026-08-27

**Authors:** Özge Selin Özen Sekmek, Gülsüm Zuhal Kamış, Bedirhan Şenol, Özgecan Özgün Erol, Mustafa Uğurlu

**Affiliations:** 1 Department of Psychiatry, Sincan Teaching and Research Hospital, Ankara, Türkiye; 2 Department of Psychiatry, Ankara Bilkent City Hospital, Ankara, Türkiye; 3 Department of Psychiatry, Faculty of Medicine, Ankara Yıldırım Beyazıt University, Ankara, Türkiye

**Keywords:** metacognition, psychosis, reliability and validity, schizophrenia, schizophrenia spectrum and other psychotic disorders

## Abstract

**Background:**

Metacognition refers to the ability to recognize one’s own and others’ feelings and thoughts and to utilize this information to develop and understanding of oneself and others. Given that metacognitive deficits are considered a key treatment target in schizophrenia spectrum disorder, the availability of reliable tools to measure these skills is essential. As an interviewer-rated scale, the Metacognition Assessment Scale-Abbreviated (MAS-A) offers advantages over self-report scales in assessing metacognitive capacity. This study aims to evaluate the validity and reliability of the Turkish version of MAS-A for clinical assessment of metacognitve skills.

**Methods:**

MAS-A was administered to 70 psychotic patients. Interrater reliability and compliance were assessed using intraclass correlation coefficients. A two-way mixed model was employed for intraclass correlation coefficients analyses. Participants were also administered: Metacognitions Questionnare-30, Beck Cognitive Insight Scale, Reading the Mind in the Eyes Test, Facial Emotion Identification and Discrimination Tests for convergent validity analysis; Positive and Negative Syndrome Scale for assessing psychopathology; Global Assessment of Functioning Scale for assessing functionality. These scales, along with the related subscales of MAS-A, were examined through correlation and hierarchical regression analyses.

**Results:**

Intraclass correlation coefficients for MAS-A total and subscale scores ranged between 0.877 to 0.968 (*p* < 0.001 for each). The Metacognitions Questionnare-30, cognitive insight scale, social cognition scales, symptomatology assessments, and functionality scale are associated with MAS-A, though the strength of these relationships is relatively low.

**Conclusion:**

The Turkish form of MAS-A has been found to be valid and reliable for patients with psychotic disorders.

## 1 Introduction

Metacognition can be defined as the ability to understand and reflect on the mental states of both oneself and others (Moritz and Lysaker, 2018). Deficits in metacognitive capacity have also been identified in psychotic disorders (Lysaker et al., 2007, 2011; Klein and Pinkham, 2020). It has been observed that metacognitive deficits in schizophrenia cause difficulties in integrating information and this also affects insight (Lysaker et al., 2019; Lungu et al., 2023). Lack of insight causes decreased compliance with treatment, increased number of relapses, depression and decreased social functioning (Lysaker, Gumley et al., 2013; Nicolò et al., 2012). Therefore, the improvement of metacognitive skills is an important goal in treatment (Lysaker et al., 2020b; Igra et al., 2022; Vohs et al., 2014). The improved metacognitive skills may increase the patient’s functionality in long-term follow-up (Moritz, 2019).

There is a substantial need for assessment methods that accurately and reliably measure metacognitive abilities. Numerous self-report instruments have been developed to assess metacognition in both clinical and non-clinical populations (Wright et al., 2019; Martiadis et al., 2023; Varadharajan et al., 2026; Yılmazer et al., 2024). Some of these, like the Metacognitions Questionnaire-30 (MCQ-30), assess cognitive beliefs associated with various psychiatric disorders such as anxiety disorders and obsessive-compulsive disorder, rather than metacognitive skills themselves. Other self-report scales that measure metacognitive skills also have limitations due to their nature as self-report scales (Palmer-Cooper et al., 2023). Completing these measures requires individuals to understand, evaluate, and report on their own mental processes, relying on the very metacognitive abilities being assessed (Bröcker et al., 2017; Hausberg et al., 2012). Consequently, individuals with metacognitive impairments may have difficulty accurately evaluating and reporting their own thoughts and behaviors, limiting the validity of self-report measures in this population (Brüne, 2014; Frith, 1992; Müller et al., 2022; Kilicoglu et al., 2024).

Semi-structured methods represent an alternative approach to assessing metacognition (Semerari et al., 2012). By using open-ended questions, these methods enable participants to describe their thoughts, emotional experiences, and perspectives in greater depth, providing a richer understanding of their metacognitive processes (Takemura et al., 2005). Compared with self-report measures, the flexible nature of semi-structured assessments allows researchers to capture individual differences more comprehensively, making them particularly valuable for evaluating metacognition (Lysaker et al., 2005).

For this purpose, the Metacognition Assessment Scale-Abbreviated (MAS-A) offers significant advantages in evaluating metacognition in schizophrenia patients (Lysaker et al., 2005; Semerari et al., 2003). The developers of scale focused on ‘synthetic metacognition’ and divided metacognition into 4 separate areas: (1) Self-Reflectivity (S); (2) Awareness of Others Mind (O); (3) Decentration (D); (4) Mastery (M). S, can be defined as realizing that one’s own mind is a separate representation from others, being able to describe one’s emotions and mental processes, being able to realize one’s thoughts are fallible, being able to distinguish between dreams and reality and connect emotions and thoughts causally. O, can be defined as the ability to understand that others have a separate mental world from one’s own, to casually connect their feelings and thoughts, and to holistically evaluate their mental world, including its causes and consequences. D, can be defined as the ability to not see oneself in the center of world, to understand that other people have a separate world and occupations, and to consider reasonable that others may think differently even the opposite of one’s own. M, can be defined as being able to find healthy ways of coping with the difficulties encountered in life, recognizing the fallibility of coping strategies with their own and others’ situations and developing them (Lysaker et al., 2005).

The MAS-A has been widely used in research and in the evaluation of treatment outcomes across different languages and cultural settings (de Jong et al., 2019; Hasson-Ohayon et al., 2024; Lysaker et al., 2005, 2010). Although the MAS-A has previously been used in a Turkish study (Aydin et al., 2016), its validity and reliability have not yet been evaluated in a Turkish population. Currently, there is no validated or gold standard interviewer-scored tool in Turkish that can assess metacognitive skills in psychotic disorders.

In this study, we aimed to address a gap in our understanding of metacognitive processes in Turkish patients with psychotic disorders by evaluating the validity and reliability of the MAS-A in Turkish. Furthermore, we hope that testing the validity and reliability of an international scale across different cultures and languages can increase the generalizability of studies conducted with this scale. Our hypotheses were as follows: (1) The MAS-A S subscale would exhibit a weak positive correlation with the Cognitive Self-Consciousness subscale of the Metacognitions Questionnaire-30 (MCQ-30), highlighting the distinction between metacognitive beliefs and metacognitive abilities. (2) The S subscale was expected to correlate positively with the Self-Reflectiveness subscale and negatively with the Self-Certainty subscale of the Beck Cognitive Insight Scale (BCIS). (3) The MAS-A O and D subscales were anticipated to show positive associations with social cognitive performance, while all MAS-A subscales were expected to relate to symptom severity (James et al., 2017; McLeod et al., 2014; Özen Sekmek et al., 2025). (4) MAS-A scores, especially the M subscale, were hypothesized to be positively linked to functioning and weakly negatively associated with metacognitive beliefs as measured by the MCQ-30 (Wright et al., 2019). (5) The Turkish version of the MAS-A was expected to demonstrate high inter-rater reliability. (6) The MAS-A is expected to be suitable for use in measuring metacognitive skills across different cultures and languages.

## 2 Methods

### 2.1 Participants

This study was conducted in Ankara City Hospital Psychiatry Clinic between 17/08/2022 to 17/03/2023. Ethics committee approval was obtained from Ankara City Hospital No. 1 Clinical Research Ethics Committee on 17/08/2022 with the number E1/2801/2022. Patients with any psychotic diagnosis, whether hospitalized in a psychiatric ward or applied to outpatient clinic, who met the inclusion criteria and provided informed consent, were consecutively included in this study.

This study included 70 patients with psychotic disorders (brief psychotic disorder, schizophreniform disorder, schizophrenia, schizoaffective disorder, delusional disorder, psychotic mania and psychotic depression) according to Diagnostic and Statistical Manual of Mental Disorders-5th Edition (DSM-5) (American Psychiatric Association, 2022) diagnostic criteria, between the ages of 18 to 65. Patients with comorbid conditions that could impair cognitive function (such as mental retardation, dementia, delirium or stroke), as well as those with active substance use or substance-related psychotic disorders, were excluded from this study. Informed consent was obtained from both the patients and their legal guardians, and their sociodemographic data were recorded. The diagnosis of patients was clarified according to the Structured Clinical Interview for DSM-5 Disorders (SCID-5) (First et al., 2016).

### 2.2 Procedure

In order to evaluate metacognitive abilities according to MAS-A, Indiana Psychiatric Illness Interview (IPII) was conducted with patients. IPII (Lysaker et al., 2002) is a semi-structured interview model that includes questions about an individual’s life story, how they cope with problems they encounter, and how psychiatric impacts their life. For this study, with permission and assistance from the scale developers, the MAS-A Codebook (Lysaker et al., 2005) and the IPII semi-structured interview questions were translated into Turkish. After translation, they were back-translated into English to minimize potential distortions in meaning. Each researcher participating in the study received training, utilizing both the IPII codebook and sample cases sent by Lysaker via e-mail.

IPII interviews were conducted with participants by three researchers (Ö. S.Ö. S., Ö.Ö. E., B.Ş.) each interview lasted 40–50 min and was audio recorded. For each IPII interview, 2 of the 3 interviewers participating in this study were present in interview room and one researcher conducted the interview, respectively. Then, the audio recordings were reviewed separately by these three researchers to assess inter-rater reliability, and metacognitive domains were scored according to MAS-A. For each interview, one of the three researchers was blinded. Apart from the two interviewers who entered the interview in turn, the third interviewer listened to the audio recordings blindly. At the time of the research, the three reviewing the audio recordings were psychiatry residents, while the other two researchers included in the study were psychiatry specialists and associate professors of psychiatry.

The Positive and Negative Syndrome Scale (PANSS) was applied to score psychopathology. To evaluate the validity of scale, MCQ-30, BCIS, Reading the Mind in the Eyes Test (RMET), Facial Emotion Identification Test (FEIT), Facial Emotion Discrimination Test (FEDT) and GAS were applied by the same researcher (Ö. S.Ö. S).

In the validity measurements, the relationships between: (1) the MAS-A S subscale with BCIS-SR and MCQ-SC subscales, (2) the MAS-A O and D subscales with RMET, FEIT and FEDT (social cognition) scales, (3) the MAS-A M subscale with GAS and MCQ-30 subscales were examined using hierarchical regression.

In reliability measurements, inter-rater reliability was used since MAS-A is a clinician-rated scale. It was essential to evaluate whether different clinicians would provide similar scores.

### 2.3 Measures

#### 2.3.1 Structured clinical interview for DSM-5 disorders (SCID-5)

It is a structured interview developed to identify psychiatric disorders according to DSM-5 diagnostic criteria (First et al., 2016). In this structured interview, 32 diagnostic categories are evaluated with detailed diagnostic criteria and 16 diagnostic categories are evaluated only with exploratory questions.

It was adapted into Turkish by Elbir et al. (2019). Diagnostic agreement rates and kappa coefficients for diagnoses were 99.4 and 0.93% for schizophrenia; 99.4 and 0.96% for bipolar I disorder; 94.5 and 0.89% for major depressive disorder; 98.3 and 0.84% for panic disorder; 96.2 and 0.89% for generalized anxiety disorder; 98.3 and 0.87% for obsessive-compulsive disorder; 98.9 and 0.89% for post-traumatic stress disorder; 99.4 and 0.96% for alcohol use disorder; and 100.0 and 1.00% for attention deficit and hyperactivity disorder, respectively.

#### 2.3.2 Positive and Negative Syndrome Scale (PANSS)

It was developed to assess the symptom severity of schizophrenia patients in terms of positive, negative and general psychopathology (Kay et al., 1987). This scale is scored by the interviewer. It has three subscales. Positive symptomatology subscale consists of 7 items, negative symptomatology subscale consists of 7 items and general psychopathology scale consists of 16 items. For each item, the lowest score is 1 and the highest score is 7. The total score is obtained by summing these scores. High scores indicate high levels of psychopathology in relevant areas.

Its Turkish validity and reliability study was conducted by Kostakoğlu et al. (1999). Cronbach alpha values of the positive, negative syndrome and general psychopathology subscales were found to be 0.75, 0.77 and 0.71, respectively.

#### 2.3.3 Metacognitions questionnaire-30 (MCQ-30)

It was developed to assess positive and negative metacognitive beliefs (Cartwright-Hatton and Wells, 1997; Wells and Cartwright-Hatton, 2004). It is a self-report scale and consists of a 5-factor structure and 30 questions including ‘Positive beliefs about worry’ (PB); ‘Negative beliefs about the uncontrollability and corresponding danger of worry’ (UD); ‘Cognitive confidence’ (CC); ‘Beliefs about need to control thoughts’ (NCT) and ‘Cognitive self-consciousness’ (SC). The items of the scale include sentences about cognitive struggles and experiences such as intrusive thoughts and anxiety. It is a four-point likert-type scale (the lowest 1 strongly disagree and the highest 4 strongly agree). The total score is obtained by summing these scores. A high score in the relevant subscale indicates a high level of pathological metacognitive beliefs.

Turkish validity and reliability study was conducted by Tosun and Irak (2008). The Cronbach Alpha reliability coefficient of the MCQ-30 is 0.86, as well as 0.72 for the first half of the scale (odd-numbered items) and 0.79 for the second half (even-numbered items).

#### 2.3.4 Beck Cognitive Insight Scale (BCIS)

It was developed to assess cognitive insight (Beck et al., 2004). It is a self-report scale. The scale consists of 2 sub-dimensions “Self-Reflectiveness” (BCIS-SR) and “Self-Certainty” (BCIS-SC); and 15 items. The BCIS-SR subscale is assessed as the person having objective opinions and taking other people’s feedback into account; the BCIS-SC subscale is assessed as the person jumping to conclusions quickly, being closed to changing one’s thoughts and being unwilling to take feedback into account. Each 15-item is scored by the participant with a minimum score of 0 (strongly disagree) and a maximum score of 3 (strongly agree). The total score is obtained by subtracting the BCIS-SC subscale score from the BCIS-SR subscale score. A high score on the scale predicts high cognitive insight.

The Turkish validity and reliability study was conducted by Aslan et al. (2005). The Cronbach Alpha reliability coefficient for BCIS-SR is 0.56 and for BCIS-SC is 0.50.

#### 2.3.5 Reading the mind in the eyes test (RMET)

It was developed to assess theory of mind (ToM) and emotion recognition (ER) skills (Baron-Cohen et al., 2001). The original form of the scale includes 36 photographs around the eyes. In the Turkish adaptation, there are 32 photographs of the eye, the first of which is a test question. For each picture, there are 4 options expressing the mental state of each picture. Participants are asked to select the option that best expresses mental state in the picture. The score obtained from the test is determined by summing number of correct answers. A high score on the test is a predictor of good ER and ToM skills.

The Turkish validity and reliability study conducted by Yıldırım et al. (2011). The mean value of the number of correct responses was 24.46 (SD = 3.44) for the test and 24.13 (SD = 4.36) for the re-test. After excluding items 19 and 21 which failed to show reliability, the mean correct response rates were 23.64 (SD = 3.38) and 23.40 (SD = 4.32), respectively.

#### 2.3.6 Facial emotion identification (FEIT) and discrimination (FEDT) tests

These were developed to assess ER skills (Kerr and Neale, 1993). Ekman and Friesen and Izard’s face photographs were used in these scales. FEIT: There are 19 black and white face photographs and six main emotion options for each photograph. The participant is asked to mark the option that best describes the emotion in photograph. A score of 1 is given for correct answers and 0 for incorrect answers. FEDT: The participant is asked to analyze the emotion expressed on the face of 30 pairs of black and white photographs of faces and evaluate whether this emotion is the same or different. If the answer is correct, 1 point is given and if the answer is wrong, 0 points are given. The total score is obtained by summing these scores. High scores on these scales predict good ER skills.

Turkish validity and reliability study was conducted by Erol et al. (2009). Test–retest reliability coefficient was 0.90 for FEI and 0.70 for FED in patient group; 0.84 for FEI and 0.93 for FED in control group, respectively. When evaluated individually, there were no statistically significant differences between first and last performances for each test item. Kappa values for FEIT items were between 0.61–0.90 in patient group, and 0.59–0.88 in control group, respectively. Kappa values for FEDT items were between 0.60–1.00 in patient group, and 0.58–1.00 in control group, respectively.

#### 2.3.7 Global Assessment of Functioning Scale (GAS)

It was developed to evaluate a person’s psychological, social and occupational functionality (Endicott et al., 1976). This scale does not consider functioning due to physical (or environmental) limitations. It is scored by the researcher, and a higher indicates better functionality.

#### 2.3.8 Metacognition Assessment Scale-Abbreviated (MAS-A)

It was developed to assess metacognitive skills (Lysaker et al., 2005; Semerari et al., 2003). The scale is scored by the researcher following a semi-structured IPII interview with participants. It includes four distinct subscales: each consisting of hierarchical levels of increasingly complex skills, with each subscale scored independently. A score of 1 point is given for each level that participant fully meets, 0.5 points for the level that participant does not fully meet, and 0 points for the level that participant does not meet at all. For S, O and D subscales: the participant must have scored 0.5 or 1 point on previous level in order to achieve next level. A maximum of 2 times 0.5 points can be given in one subscale. However, in M subscale, there is no requirement to score points from the previous levels in order to achieve the next levels. S subscale can be scored 0 to 9; O subscale 0 to 7; D subscale 0 to 3; M subscale 0 to 9. Higher subscale scores predict better metacognitive capacity (Lysaker et al., 2005).

### 2.4 Statistical analysis

Data from the study conducted for the German adaptation of the scale were used to determine the sample size (Bröcker et al., 2017). The intraclass correlation coefficient/inter-rater reliability coefficients of the scale were examined with the Gpower 3.1.9.7 program. It was calculated that the sample size should be at least 62 with an effect size of 0.51, a confidence interval of 95%, and a power of 80% (Faul et al., 2009).

Statistical analyses were performed using IBM SPSS Statistics 26 for Windows and JAMOVI programmes. The Chi-Square test was used for comparisons between categorical variables, which are expressed as numbers and percentages. Continuous variables are presented as mean±standard deviation. In comparisons between two groups, Student’s T-test was used for pairwise comparisons according to distribution characteristics. One-way ANOVA was used for comparisons between three or more groups. Bonferroni correction was used in posthoc analyses. Interrater reliability and compliance were assessed by Intraclass Correlation Coefficient (ICC).

We used the JAMOVI to model ICC analyses. A two-way mixed model was used in ICC analyses. Pearson correlation analysis was performed to assess convergent and divergent validity, and hierarchical regression analysis was performed to further examine which constructs we examined were related to the MAS-A subscales. The threshold value for statistical significance was accepted as *p* < 0.05.

## 3 Results

Sociodemographic and clinical characteristics of participants have been shown in Table 1.

**Table 1:** Sociodemographic and clinical features of the participants.

Sociodemographic and clinical features		Participants
Age (Mean±SD)		34,8 ± 12
Gender *n* (%)	Female	31(%44)
	Male	39(%56)
Education status	Primary school	11(%15.7)
	Middle school	10(%14.3)
	High school	33(%47.1)
	Associate degree	3(%4.3)
	University student	5(%7.1)
	University	8(%11.4)
Marital status	Single	55(%78.6)
	Married	15(%21.4)
Diagnosis	Schizophrenia	42(%60)
	Schizophreniform disorder	4(%5.7)
	Acute brief psychosis	4(%5.7)
	Psychotic depression	4(%5.7)
	Psychotic mania	9(%12.9)
	Schizoaffective disorder	3(%4.3)
	Delusional disorder	4(%5.7)

Categorical variables are given as number (percent) [n(%)], numerical variables are given as Mean+Standard deviation (Mean+SD).

Scale points of participants have been shown in Table 2.

**Table 2:** Scale points of the participants.

		(Mean±SD)
PANSS points	PANSS positive	22 ± 6,5
	PANSS negative	21.5 ± 6.8
	PANSS general	36.7 ± 6.7
	PANSS total	80.4 ± 14
MAS-A points	S	4.8 ± 1.8
	O	3.9 ± 1.3
	D	1.2 ± 0.8
	M	3.5 ± 1.7
	Total	13.4 ± 4.6
MCQ-30		72.8 ± 14.3
RMET		18.3 ± 4.8
FEIT		10.5 ± 3.4
FEDT		23.6 ± 4.2
BCIS		0.7 ± 6.4
GAS		21.9 ± 9.4

PANSS, Positive and Negative Syndrome Scale; MAS-A, Metacognition Assessment Scale-Abbreviated; S, self-reflectivity; O, awareness of others mind; D, decentration; M, mastery; MCQ-30, metacognitions questionnare-30; BCIS, Beck Cognitive Insight Scale; RMET, reading the mind in the eyes test; FEIT, facial emotion identification test; FEDT, facial emotion discrimination test; GAS, Global Assessment of Functioning Scale.

### 3.1 Reliability and validity analysis

**Figure 1:**
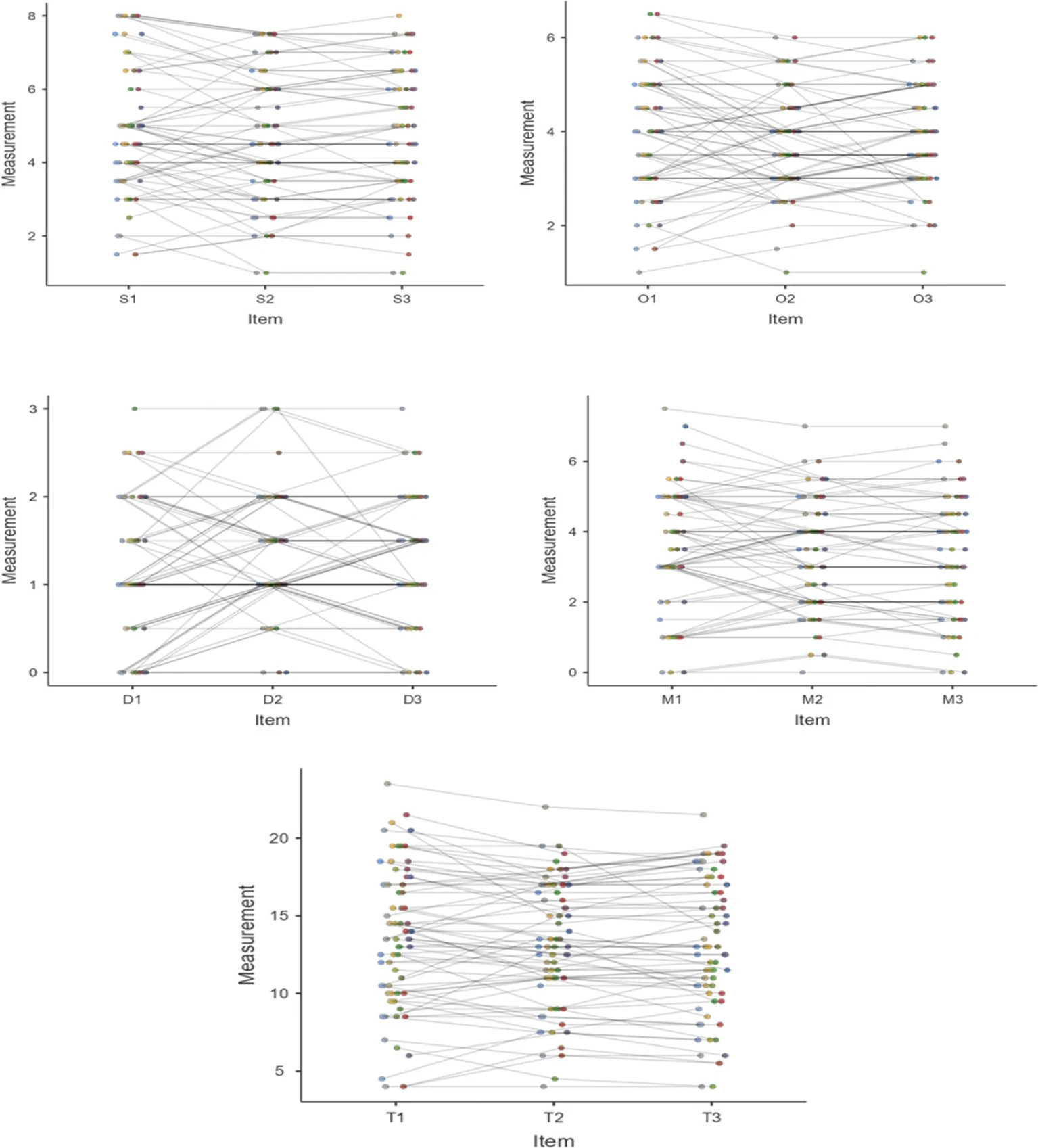
Intraclass correlation coefficient for MAS-A. Evaluators are represented by the numbers 1, 2, and 3; S, self-reflectivity; O, awareness of others mind; D, decentration; M, mastery; T, total score.

According to ICC analyzes for three observers: ICC values of MAS-A total and subscale scores were between 0.877 to 0.968 (*p* < 0.001 for each) (Table 3 and Figure 1).

**Table 3:** Intraclass correlation coefficient for MAS-A.

		95% Confidence interval			
MAS-A	ICC	Lower bound	Upper bound	Value	Sig
S	0.958	0.938	0.973	23.87	**<0.001**
O	0.914	0.873	0.944	11.688	
D	0.877	0.817	0.92	8.14	
M	0.956	0.934	0.971	22.557	
Total	0.968	0.953	0.979	31.662	

MAS-A, Metacognition Assessment Scale-Abbreviated; S, self-reflectivity; O, awareness of others mind; D, decentration; M, mastery; ICC, intraclass correlation coefficient.

Correlations between subscales were *r* = 0.473–0.638 (*p* < 0.001); correlations between subscales and the total score were *r* = 0.711–0.882 (*p* < 0.001). And Cronbach’s alpha coefficient was calculated as 0.807 for the scale.

To evaluate the validity of the scale, correlations between MAS-A and BCIS, RMET, FEIT, FEDT, GAS scales were examined. There was no significant correlation between MAS-A and MCQ-30 scores (*p* > 0.05). D subscale positively weak correlated with BCIS total score (*r* = 0.239, *p* < 0.05) and BCIS-SR subscale (*r* = 0.295, *p* < 0.05). There were moderate positive correlations between MAS-A total score and all subscales with RMET (*r* = 0.355 for S, *r* = 0.329 for O, *r* = 0.320 for D, 0.446 for M, *p* < 0.001; *r* = 0.449 for MAS-A total score *p* < 0.001). There were weak positive correlations between MAS-A total score and S, O, M subscales with FEDT (*r* = 0.279 for S; *r* = 0.265 for O; *r* = 0.268 for M; *r* = 0.300 for MAS-A total score *p* < 0.05). A borderline significant positive correlation was detected between FEIT and M subscale (*r* = 0.232; *p* = 0.053) (Table 4). MAS-A total and all subscale scores positively moderate correlated with GAS (*r* = 0.305 to 0.436; *p* < 0.01 to 0.001) (Table 4).

**Table 4:** Correlations between MAS-A and other scales.

Scales		S	O	D	M	MAS-A
RMET	*r*	**0.355**	**0.329**	**0.320**	**0.446**	**0.449**
	*p*	**0.003**	**0.005**	**0.007**	**<0.001**	**<0.001**
FEIT	*r*	0.029	0.117	0.029	*0.232*	0.135
	*p*	0.815	0.336	0.814	*0.053*	0.264
FEDT	*r*	**0.279**	**0.265**	0.106	**0.268**	**0.300**
	*p*	**0.019**	**0.026**	0.384	**0.025**	**0.012**
BCIS-SR	*r*	−0.079	−0.067	**0.295**	0.135	0.051
	*p*	0.517	0.580	**0.013**	0.265	0.676
BCIS-SC	*r*	−0.062	−0.068	−0.021	−0.119	−0.091
	*p*	0.609	0.574	0.860	0.325	0.452
MCQ-30 PB	*r*	−0.206	−0.115	0.085	−0.155	−0.155
	*p*	0.086	0.341	0.484	0.201	0.199
MCQ-30 UD	*r*	−0.180	**−0.271**	−0.016	−0.095	−0.184
	*p*	0.137	**0.023**	0.893	0.434	0.128
MCQ-30 CC	*r*	0.005	*−0.220*	−0.067	0.040	−0.057
	*p*	0.970	*0.067*	0.583	0.745	0.641
MCQ-30 NCT	*r*	0.033	−0.099	0.071	0.135	0.047
	*p*	0.784	0.415	0.557	0.264	0.697
MCQ-30 SC	*r*	0.182	0.158	*0.217*	0.137	0.202
	*p*	0.132	0.191	*0.071*	0.259	0.094
GAS	*r*	**0.334**	**0.409**	**0.305**	**0.377**	**0.436**
	*p*	**0.005**	**<0.001**	**0.010**	**0.001**	**<0.001**

Pearson correlation analysis was used. *P* < 0.05 was accepted significant. MAS-A, Metacognition Assessment Scale-Abbreviated; S, self-reflectivity; O, awareness of others mind; D, decentration; M, mastery; RMET, reading the mind in the eyes test; FEIT, facial emotion identification test, FEDT, facial emotion discrimination test; BCIS, Beck Cognitive Insight Scale; BCIS-SR, self-reflectiveness; BCIS-SC, self-certainty; MCQ-30, metacognitions questionnare-30; MCQ-30 PB, positive beliefs about worry; MCQ-30 UD: Negative beliefs about the uncontrollability and corresponding danger of worry; MCQ-30 CC, cognitive confidence; MCQ-30 NCT, beliefs about need to control thoughts: MCQ-30 SC, cognitive self-consciousness; GAS, Global Assessment of Functioning Scale.

When examining the correlation between MAS-A and PANSS scores, the MAS-A total score and all subscales demonstrated a negative moderate correlation with the PANSS negative psychopathology score (S: *r* = −0.449; *p* < 0.001, O: −0.648; *p* < 0.001, D: −0.523; *p* < 0.001, M: −0.472; *p* < 0.001, T: −0.619; *p* < 0.001). A weak negative correlation was found between M subscale and PANSS positive psychopathology score (*r* = −0.253; *p* < 0.035). No significant relationship was found between the PANSS Positive subscale and the other subscales and total score of MAS-A. No significant correlation was found between the PANSS General Psychopathology subscale and the MAS-A scale and subscale scores.

According to the theoretical model and the results of correlation analysis, hierarchical regression analysis was performed for the MAS-A subscales and total score with factors that may be related. It was theorized that the following subscales are interrelated: RMET, GAS, FEDT, MCQ-SC and BCIS-SR with S; RMET, FEDT, GAS, MCQ-UD, MCQ-CC with O; RMET, GAS, BCIS-SR and MCQ-CC with D; RMET, GAS and FEDT subscales with M; MAS-A total score and all above scales. According to the results of hierarchical regression analysis, RMET was positively related with all of the subscales of MAS-A: S scale (*p* = 0.003; *B* = 0.130), O subscale (*p* = 0.035; *B* = 0.063), D subscale (*p* = 0.007; *B* = 0.051), M subscale (*p* < 0.001; *B* = 0.160) and MAS-A total (*p* < 0.001; *B* = 0.430). There was a positive relationship between S subscale and GAS (*p* < 0.022; *B* = 0.050). There was a negative relationship between O subscale and MCQ-UD (*p* < 0.022; *B* = -0.081), a positive relationship between O subscale and GAS (*p* < 0.001; *B* = 0.055).

There was a positive relationship between D subscale and BCIS-SR (*p* < 0.012; *B* = 0.045), a positive relationship between D subscale and GAS (*p* < 0.023; *B* = 0.210). There was a positive relationship between M subscale and GAS (*p* < 0.009; *B* = 0.053) (Table 5).

**Table 5:** Hierarchical regression analysis of subscales of the MAS-A.

Self-reflectivity								Understanding other’s mind							
Model		Adjusted R^2^	*B*	Std. error	Beta	*t*	*p*	Model		Adjusted R^2^	B	Std. error	Beta	*t*	*p*
1	RMET	0.113	0.13	0.042	0.355	3.128	**0.003**	1	GAS	0.155	0.057	0.015	0.409	3.701	**<0.001**
2	RMET	0.168	0.107	0.042	0.292	2.584	**0.012**	2	GAS	0.208	0.055	0.015	0.398	3.708	**<0.001**
	GAS		0.05	0.021	0.265	2.345	**0.022**		MCQ-UD		−0.081	0.034	−0.252	−2.352	**0.022**
3	RMET	0.160	0.085	0.056	0.231	1.527	0.132	3	GAS	0.249	0.048	0.015	0.344	3.198	**0.002**
	GAS		0.05	0.021	0.268	2.358	**0.021**		MCQ-UD		−0.077	0.034	−0.24	−2.291	**0.025**
	FEDT		0.038	0.063	0.091	0.613	0.542		RMET		0.063	0.029	0.232	2.156	**0.035**
4	RMET	0.153	0.088	0.056	0.239	1.57	0.121	4	GAS	0.243	0.048	0.015	0.347	3.211	**0.002**
	GAS		0.05	0.022	0.264	2.315	**0.024**		MCQ-UD		−0.076	0.034	−0.238	−2.266	**0.027**
	FEDT		0.035	0.063	0.083	0.559	0.578		RMET		0.045	0.039	0.167	1.165	0.248
	BCIS-SR		−0.027	0.04	−0.075	−0.676	0.502		FEDT		0.03	0.044	0.096	0.681	0.498
5	RMET	0.169	0.095	0.056	0.258	1.703	0.093	5	GAS	0.238	0.047	0.015	0.336	3.08	**0.003**
	GAS		0.047	0.021	0.248	2.18	**0.033**		MCQ-UD		−0.067	0.036	−0.209	−1.863	0.067
	FEDT		0.022	0.063	0.052	0.346	0.731		RMET		0.045	0.039	0.167	1.159	0.251
	BCIS-SR		−0.044	0.042	−0.121	−1.057	0.294		FEDT		0.029	0.044	0.094	0.669	0.506
	MCQ-SC		0.085	0.056	0.173	1.504	0.137		MCQ-CC		−0.027	0.036	−0.084	−0.741	0.462

Decentration								Mastery							
Model		Adjusted R^2^	B	Std. error	Beta	*t*	*p*			Adjusted R^2^	B	Std. error	Beta	*t*	*p*
1	RMET	0.089	0.051	0.018	0.32	2.783	**0.007**	1	RMET	0.187	0.16	0.039	0.446	4.113	**<0.001**
2	RMET	0.159	0.05	0.018	0.31	2.81	**0.006**	2	RMET	0.255	0.135	0.038	0.379	3.541	**0.001**
	BCIS-SR		0.045	0.018	0.285	2.582	**0.012**		GAS		0.053	0.02	0.287	2.687	**0.009**
3	RMET	0.211	0.04	0.018	0.25	2.266	**0.027**	3	RMET	0.245	0.144	0.051	0.403	2.814	**0.006**
	BCIS-SR		0.047	0.017	0.296	2.762	**0.007**		GAS		0.052	0.02	0.286	2.655	**0.01**
	GAS		0.021	0.009	0.256	2.325	**0.023**		FEDT		−0.015	0.058	−0.037	−0.262	0.794
4	RMET	0.213	0.04	0.018	0.248	2.249	0.028								
	BCIS-SR		0.042	0.018	0.266	2.403	**0.019**								
	GAS		0.02	0.009	0.245	2.222	**0.03**								
	MCQ-SC		0.025	0.024	0.118	1.067	0.29								

MAS-A, Metacognition Assessment Scale-Abbreviated; MCQ, metacognitions questionnare; MCQ-SC, MCQ cognitive self-consciousness; MCQ-CC, MCQ cognitive confidence; MCQ-UD, MCQ UD, Negative beliefs about the uncontrollability and corresponding danger of worry. BCIS: Beck Cognitive Insight Scale; BCIS-SR, self-reflectiveness; RMET, reading the mind in the eyes test; FEDT: Facial Emotion Discrimination Test; GAS, Global Assessment of Functioning Scale. B, standardized coefficients beta: standardized coefficients.

There was a positive relationship between MAS-A total and RMET (*p* < 0.001; *B* = 0.430), a positive relationship between MAS-A total and GAS (*p* = 0.001; *B* = 0.171), a negative relationship between MAS-A total and BCIS-SC (*p* = 0.042; *B* = -0.277) (Table 6)

**Table 6:** Hierarchical regression analysis of the MAS-A.

Scales		Adjusted R^2^	Unstandardized coefficients		Standardized coefficients	*t*	Sig.
			B	Std. error	Beta		
MAS-A Total	RMET	0.190	0.430	0.105	0.449	2.798	**<0.001**
	RMET	0.296	0.351	0.099	0.367	3.530	**0.001**
	GAS		0.171	0.051	0.349	3.358	**0.001**
	RMET	0.370	0.310	0.097	0.324	3.187	**0.002**
	GAS		0.152	0.051	0.310	3.004	**0.004**
	MCQ-PB		−0.204	0.608	−0.211	−0.335	0.739
	MCQ-UD		−0.573	0.617	−0.504	−0.929	0.357
	MCQ-CC		0.065	0.594	−0.058	−0.110	0.913
	MCQ-NCT		0.186	0.628	0.165	0.297	0.768
	MCQ-SC		0.275	0.643	0.216	0.428	0.670
	MCQ-30 total		0.102	0.607	0.316	0.168	0.867
	RMET	0.392	0.328	0.137	0.343	2.392	**0.020**
	GAS		0.139	0.051	0.284	2.732	**0.008**
	MCQ-PB		0.148	0.628	0.153	0.236	0.814
	MCQ-UD		−0.199	0.641	−0.175	−0.331	0.757
	MCQ-CC		0.224	0.609	0.198	0.367	0.715
	MCQ-NCT		0.620	0.655	0.550	0.948	0.347
	MCQ-SC		0.775	0.679	0.608	1.141	0.259
	MCQ-30 total		−0.286	0.633	−0.885	−0.451	0.653
	FEIT		−0.190	0.172	−0.138	−1.108	0.272
	FEDT		0.076	0.151	0.069	0.502	0.618
	BCIS-SR		0.048	0.113	0.050	0.422	0.675
	BCIS-SC		−0.277	0.134	−0.234	−2.076	**0.042**

MAS-A, Metacognition Assessment Scale-Abbreviated; MCQ-30, Metacognitions Questionnare-30; PB, positive beliefs about worry; UD, negative beliefs about the uncontrollability and corresponding danger of worry; CC, cognitive confidence; NCT, beliefs about need to control thoughts: SC, cognitive self-consciousness; BCIS, Beck Cognitive Insight Scale; BCIS-SR, self-reflectiveness; BCIS-SC, self-certainty; BCIS-T, BCIS total RMET, reading the mind in the eyes test; FEIT, facial emotion identification test, FEDT, facial emotion discrimination test; GAS, Global Assessment of Functioning Scale.

## 4 Discussion

This study on the adaptation of the MAS-A scale into Turkish, along with its validity and reliability, found that MAS-A is valid and reliable for use in a clinical population diagnosed with psychotic disorders in Türkiye. ICC, inter-rater reliability and internal consistency were found to be high. These findings are consistent with studies showing high inter-rater reliability and consistency for MAS-A across different cultures (Bröcker et al., 2023; Lysaker et al., 2020a).

While previous studies used cohorts with milder symptom severity (Bröcker et al., 2017; Lysaker et al., 2020a), this study includes patients with more severe psychopathology. This indicates that MAS-A is reliable and has high inter-rater reliability in patients with severe psychopathology. The inclusion of a large sample comprising different disease groups and varying severity levels shows that the scale can be reliably used across various patient populations with psychosis.

In internal consistency measurement, it was observed that MAS-A subscales were well correlated with each other and with MAS-A total score. This is in line with other studies showing good internal consistency (Lysaker et al., 2007). It also suggests that MAS-A total score can be used to evaluate metacognition (Bröcker et al., 2023).

In terms of convergent validity, in correlation analysis, no significant relationship was found between MCQ-30 and MAS-A. And in hierarchical regression analysis, a significant negative relationship was found between UD and O subscales, but the strength of this relationship is quite weak. This is consistent with studies in which clinical interview-scored scales were compared with self-report scales (Bröcker et al., 2017; Brüne, 2014; Frith, 1992). The absence of significant associations between any MAS-A subscale and the MCQ-30 provides evidence for discriminant validity. This finding suggests that the MAS-A assesses a construct distinct from that measured by the MCQ-30. MCQ-30 does not assess metacognitive capacity, but rather the content of metacognitive beliefs associated with anxiety and obsession (Wells and Cartwright-Hatton, 2004; Tosun and Irak, 2008), whereas MAS-A assesses four metacognitive abilities. Unlike the MCQ-30, MAS-A focuses on the individual’s life story, how their mental illness is reflected in their daily life, the challenges they face in interpersonal relationships, and how they cope with these difficulties (Bröcker et al., 2023). Therefore, although both scales are related to metacognition, they do not measure the same aspects (Ayala et al., 2023). In this respect, it can be said that the MAS-A offers a more global perspective on metacognition in psychotic disorders.

In this study, a positive correlation was found between the D subscale and the BCIS-SR subscale (an indicator of enhanced cognitive insight). Additionally, hierarchical regression analysis revealed a negative relationship between the MAS-A total score and the BCIS-SC subscale (an indicator of lack of insight). This aligns with studies suggesting that individuals with enhanced cognitive insight also demonstrate better metacognitive abilities. However, in these studies, the relationship between S, O and M skills with insight was also observed (Lysaker et al., 2019; Lysaker, Vohs et al., 2013). In studies with larger samples, the relationship between BCIS scores and other metacognitive skills may become more visible. The association between BCIS-SR and the D subscale may be related to their similarities in understanding, differentiating, and accepting others’ perspectives. Thus, it can be considered to use D subscale in clinical follow-up, such as the BCIS-SR subscale (Lopez-Morinigo et al., 2020), in monitoring the change in cognitive insight component, which is also the focus of Metacognitive Reflection and Insight Therapy.

In this study, all MAS-A subscales and total score correlated with RMET, which measures ToM and ER skills. In addition, both scales measuring ER (FEIT and FEDT) showed correlations with M; MAS-A total score and all subscales except D showed correlations with FEDT. This is consistent with studies showing that metacognition is related to ToM, social cognition, and mentalization (Fonagy et al., 2011; James et al., 2017).

The hierarchical regression analysis supports the fact that, M ability, which is a reflection of the ability to cope with life’s problems, has close connections with social functionality (Ferrer-Quintero et al., 2021; Lysaker et al., 2011b). The correlations between MAS-A total and subscales with RMET in hierarchical regression analysis are consistent with studies showing that ER and ToM are associated with metacognitive capacity (Lysaker et al., 2020a). Better metacognitive abilities also make it easier to understand feelings and perspective of others. It can be thought that in order to see other’s perspectives different from one’s own, it is necessary to interpret others’ perspectives and feelings correctly, and to know what others feel and think in same situation (Fonagy et al., 2002; Dimaggio et al., 2008). Additionally, the ability to evaluate others from different perspectives can make it easier to understand other’s emotions (Lysaker et al., 2014).

In hierarchical regression analysis, RMET, which is related to ToM, was found to be related to various subscales of MAS-A, while no relationship was found with FEIT and FEDT tests measuring ER, suggesting that metacognition has a closer relationship with ToM (Fonagy et al., 2011). Recent research has demonstrated that the relationship between metacognition and ToM, previously established in psychotic disorders, is also evident in individuals with depression (Demiray and Bacık Yaman, 2025). These findings suggest that the interplay between metacognitive abilities and ToM might extend beyond psychotic disorders, indicating a potential broader relevance across various psychiatric conditions. However, the low strength of relationship between ToM and MAS-A subscales also supports that these two interrelated structures are separate things.

The low correlation with the scales used in the validity analysis of the study is an expected result, as there is no gold standard scale in Turkish for measuring metacognitive skills that can be used clinically.

The results of this study shows that although metacognition is related to insight, ToM, and social cognition, it’s a separate structure from all these. It shows that MAS-A is a different metacognitive assessment tool than MCQ-30 and the Turkish form of MAS-A is a valid and reliable assessment tool.

The negative correlations between PANSS negative subscale with MAS-A total and subscale scores indicate that MAS-A is associated with symptomatology, particularly negative symptoms. Our findings are consistent with studies in literature showing that people with good metacognitive abilities exhibit lower negative symptomatology scores (Trauelsen et al., 2016) and, in some studies, their positive symptomatology scores are also lower (McLeod et al., 2014).

The correlations between GAS with MAS-A total and subscale scores indicates that MAS-A is associated with better functioning. Our findings are consistent with studies in literature showing that people with good metacognitive abilities have better functionality (Wright et al., 2019). Correlations between symptomatology and functionality may suggest that the MAS-A is a suitable scale in psychotherapy research (Bröcker et al., 2023).

This study presents several limitations that should be considered when interpreting the findings. First, the inclusion of all patients hospitalized or presenting to an outpatient clinic during the study period may have influenced the results, as the MAS-A is closely associated with psychopathology. The inability to re-administer the scale during periods of symptom remission may have restricted the assessment of the Turkish version’s reliability. Future studies incorporating repeated measures at different time points and employing longitudinal designs could address this limitation. Second, the sample included patients with psychotic depression and mania in addition to those with schizophrenia spectrum disorders. This diagnostic heterogeneity may have impacted the specificity, validity, and reliability of findings related to the schizophrenia spectrum. Restricting future samples or conducting subgroup analyses may help clarify disorder-specific results. Third, the observed high inter-rater reliability might be attributed to the fact that all evaluators were trained together and worked within a single center. Multicenter studies are warranted to more accurately assess inter-rater reliability and enhance the generalizability of findings. Fourth, the absence of a test–retest reliability assessment limits conclusions regarding the temporal stability of the scale. Incorporating test–retest measures in future research would provide valuable information on the scale’s consistency over time. Additionally, in this study, the RMET was used when examining the validity of MAS-A. Future studies may benefit from more context-sensitive measures of social cognition, as these may better reflect the contextual and integrative nature of the MAS-A. Finally, there is currently no Turkish scale that directly evaluates the four metacognitive domains assessed by the MAS-A, precluding an external validity analysis using a comparable instrument. While this is a limitation, it also highlights the need for such an assessment tool in Turkish clinical settings.

## 5 Conclusion

In conclusion, the results of this study demonstrate that metacognition is closely related to insight, ToM, and social cognition, yet it remains a distinct construct from these domains. MAS-A serves as a unique metacognitive assessment tool, distinct from MCQ-30. The Turkish version of MAS-A has been shown to be a valid and reliable assessment tool in a clinical sample with psychotic disorders. This study significantly contributes to the evaluation of metacognitive skills, highlights the importance of such assessments, and guides future research in this area.

The findings of this study highlights that MAS-A is a valid and reliable tool in Turkish for the clinical assessment of metacognitive skills, which may impact functionality and symptomatology in patients with psychotic disorders during both acute and chronic stages. It can also be used to monitor the effectiveness of metacognitive interventions.

## Data Availability

The original contributions presented in the study are included in the article/supplementary material, further inquiries can be directed to the corresponding author/s.

## Glossary

BCIS: Beck Cognitive Insight Scale
BCIS-SC: Self-Certainty
BCIS-SR: Self-Reflectiveness
CC: Cognitive confidence
D: Decentration
DSM-5: Diagnostic and Statistical Manual of Mental Disorders- 5th Edition
ER: Emotion Recognition
FEDT: Facial Emotion Discrimination Test
FEIT: Facial Emotion Identification Test
GAS: Global Assessment of Functioning Scale
IPII: Indiana Psychiatric Illness Interview
M: Mastery
MAS-A: Metacognition Assessment Scale- Abbreviated
MCQ-30: Metacognitions Questionnaire-30
NCT: Beliefs about need to control thoughts
O: Awareness of Others’ Mind
PANSS: Positive and Negative Syndrome Scale
PB: Positive beliefs about worry
RMET: Reading the Mind in the Eyes Test
S: Self- Reflectivity
SC: Cognitive self-consciousness
SCID-5: Structured Clinical Interview for DSM-5 Disorders
ToM: Theory of Mind
UD: Negative beliefs about the uncontrollability and corresponding danger of worry
